# Influence of Channel Selection and Subject’s Age on the Performance of the Single Channel EEG-Based Automatic Sleep Staging Algorithms

**DOI:** 10.3390/s23020899

**Published:** 2023-01-12

**Authors:** Waleed Nazih, Mostafa Shahin, Mohamed I. Eldesouki, Beena Ahmed

**Affiliations:** 1 College of Computer Engineering and Sciences, Prince Sattam bin Abdulaziz University, Al Kharj 11942, Saudi Arabia; 2 School of Electrical Engineering and Telecommunications, University of New South Wales, Sydney, NSW 2052, Australia

**Keywords:** sleep stage scoring, pediatric, EEG, electroencephalogram, deep learning

## Abstract

The electroencephalogram (EEG) signal is a key parameter used to identify the different sleep stages present in an overnight sleep recording. Sleep staging is crucial in the diagnosis of several sleep disorders; however, the manual annotation of the EEG signal is a costly and time-consuming process. Automatic sleep staging algorithms offer a practical and cost-effective alternative to manual sleep staging. However, due to the limited availability of EEG sleep datasets, the reliability of existing sleep staging algorithms is questionable. Furthermore, most reported experimental results have been obtained using adult EEG signals; the effectiveness of these algorithms using pediatric EEGs is unknown. In this paper, we conduct an intensive study of two state-of-the-art single-channel EEG-based sleep staging algorithms, namely DeepSleepNet and AttnSleep, using a recently released large-scale sleep dataset collected from 3984 patients, most of whom are children. The paper studies how the performance of these sleep staging algorithms varies when applied on different EEG channels and across different age groups. Furthermore, all results were analyzed within individual sleep stages to understand how each stage is affected by the choice of EEG channel and the participants’ age. The study concluded that the selection of the channel is crucial for the accuracy of the single-channel EEG-based automatic sleep staging methods. For instance, channels O1-M2 and O2-M1 performed consistently worse than other channels for both algorithms and through all age groups. The study also revealed the challenges in the automatic sleep staging of newborns and infants (1–52 weeks).

## 1. Introduction

Humans spend almost one-third of their lifetime sleeping. Healthy sleep is vital for human physical and mental health. Sleep disorders, such as insomnia, apnea, and parasomnias, affect the quality of sleep, causing medical conditions, such as depression, difficulty concentrating, and weight gain [1]. Delays in the treatment of some sleep disorders can lead to more serious diseases, such as heart disease, diabetes, and memory loss [2,3,4]. Polysomnography, or a sleep study, is commonly used to diagnose sleep disorders. A polysomnography includes various biosignals measured during eight hours of overnight sleep [5]. Electroencephalogram (EEG), which measures the brain’s electrical activity, is the main biosignal measure used in sleep staging, the time-intensive step of identifying the five different stages in the EEG signal. Sleep stage scoring is typically performed by dividing the EEG signal into epochs and assigning each epoch to one of the five sleep stages defined by the American Academy of Sleep Medicine (AASM) [6], namely wake stage (W), rapid eye movement stage (REM), and non-random eye movement stages (N1, N2, and N3). N3 is what is known as the deep sleep stage. The amount of sleep spent in the deep sleep stage is a key indicator of healthy sleep.

Manual sleep stage scoring is costly and a resource- and time-consuming process. Therefore, there is an increased interest in automatic sleep stage scoring techniques to support experts in their diagnosis. Several approaches have been proposed to automatically classify each epoch to one of the five sleep stages, including shallow classifiers, such as support vector machine [7], random forest [8], decision tree [9], as well as deep learning techniques [10], such as convolutional neural network (CNN) [11] and long short-term memory (LSTM) [12,13]. Most of the recent automatic sleep stage scoring methods rely on a signal from a single EEG channel [12,13,14,15,16,17,18,19]. A typical EEG-based sleep stage scoring method consists of pre-processing and feature extraction modules followed by the classifier module. However, recent end-to-end deep learning methods use the raw EEG signal as an input to a trainable front-end feature extraction network. The most commonly used architecture for the feature extraction network is the multi-resolution convolutional neural networks (CNNs) [18,20].

In polysomnography, multiple EEG channels are collected using a cap placed on the head, which patients find uncomfortable and disruptive. Collecting data from just a single EEG channel would be more comfortable for the patient and enable home monitoring. It would also make the collection of large datasets needed to train data-hungry algorithms for sleep stage scoring, such as deep learning methods, more feasible. Knowing which channel is the most effective is, thus, crucial. Due to the limitation of available sleep datasets in terms of the number of channels and the number and varieties of participants, most of the current work has been applied to a limited number of channels and focused only on adult sleep signals.

Children’s sleep is known to have significant variations across different age groups and within the same age group [21]. However, due to the unavailability of adequate child sleep data, very little work has been carried out on automatically analyzing child sleep [13,16,22].

Therefore, in this paper, the following questions were investigated:

- How does the type of EEG channel used impact the accuracy of automatic sleep staging?
- How does the accuracy of automatic sleep staging vary with the subject’s age?
- How effective is a model trained on adult EEG data for sleep staging on pediatric EEG signals?

To answer these questions, the performance of two state-of-the-art (SOTA) automatic sleep staging algorithms, DeepSleepNet [20] and AttnSleep [18], was compared on seven different EEG channels and over 19 age ranges between the ages of 6 days to ~58 years.

The remainder of the paper is organized as follows. Section 2 highlights recent work on automatic sleep staging. The two adopted sleep staging algorithms are summarized in Section 3 along with the utilized dataset. The experimental setup is detailed in Section 4. The results are presented in Section 5 and discussed in Section 6. Finally, the conclusion is drawn in Section 7.

## 2. Related Work

Existing sleep staging techniques can be divided into two main categories: classical (shallow) machine learning and deep learning-based. Although classical machine learning techniques are computationally inexpensive, their accuracy is highly dependent on the selection of hand-crafted features.

Deep learning techniques have recently made significant advancements in their classification capabilities. These techniques can extract features of EEG signals directly and capture the transition relationship between sleep stages. However, a considerable amount of training data is needed to train a reliable deep learning sleep staging model.

In [16], a bi-stream adversarial learning network was proposed for classifying sleep stages of pediatric data. The proposed system consists of two stream networks (i.e., Student and Teacher) in addition to a similarity function to reduce the difference between the output of the aforementioned networks. Furthermore, two datasets were used to evaluate the proposed system. The first one named NPH contains O1-M2 EEG data of 15 pediatric participants, while the second dataset is a subset of the Sleep-EDF dataset that contains Fpz-Cz EEG data of eight adult participants. The proposed systems achieved remarkable results (i.e., 80% and 91% over NPH and a subset of the Sleep-EDF, respectively) compared with other systems, although they struggled with the N1 sleep stage.

In [22], a hybrid multi-domain neural network was proposed. The proposed system used CNN and bidirectional LSTM (BLSTM) models for classifying sleep stages of pediatric EEG data. Two layers of CNN were used to model the frequency and time domain information followed by the LSTM layer to model the dependency of the data within every epoch. The proposed system was tested using a dataset of 115 female and 103 male pediatric participants with 32 EEG channels. In addition, the effect of epoch length, the number of channels to be used, and the use of frequency and time domain information was investigated. The proposed system achieved 92% accuracy but over three sleeping stages only (i.e., W, N1, and N2).

A model that utilized the CNN and recurrent neural network (RNN) was introduced in [15]. This model is composed of three layers of convolutional neural networks and two layers of recurrent neural networks. The proposed model was trained using a dataset of ECG signals of two groups of participants (i.e., controlled and uncontrolled) with a total of 71 male participants and 41 female participants. All participants were above 48 years old. The model accuracy was high in the case of three-stage classification (i.e., 86.4%) and low with five-stage classification (i.e., 74.2%). One of the drawbacks of this model is its higher computational load compared to other models.

Enhancing feature extraction from EEG signals was performed in [17]. The authors added an attention component to the CNN to learn local correlations of EEG signals. The CNN layers were followed by two layers of BLSTM to extract global correlations of sequential epochs. The sleep-EDF dataset with its two versions, 2013 and 2018, was used to evaluate the proposed model. It achieved 84.14% and 82.58% accuracy over the Fpz-Cz and Pz-Oz channels, respectively, on the 2013 version. However, less accuracy was achieved with the 2018 version.

The authors of [14] used classical machine learning algorithms to classify sleep stages. In the feature extraction phase, they extracted a set of frequency and time domain features from EEG signals. In addition, they used assigned weights for each feature to determine its importance. These weights were used to decrease the number of features from 59 to only 11. In the classification phase, decision trees, support vector machine, random forest, and backpropagation neural network were tested. The random forest classifier achieved the best accuracy over the Fpz-Cz channel from the expanded Sleep-EDF dataset. It achieved 83.56% and 82.53% using 59 and 11 features, respectively.

Similarly, time and frequency domain features were extracted from EEG signals in [23]. The authors extracted a set of 136 features and proposed a technique based on ant colony optimization (ACO) to reduce the features to only 40 features. In addition, they utilized a random forest classifier for sleep stage classification. Furthermore, they improved the classification accuracy using the hidden Markov model (HMM), which takes into account knowledge about the temporal pattern of sleep stage transitions. The proposed model was faster than RNN- and CNN-based models and less susceptible to modifications of hyperparameters, but its feature extraction process was time-consuming.

The authors of [24] used the C4-M1 channel to develop clinical decision support systems (CDSSs). The authors proposed a deep learning model that utilized CNN and transformers to classify three sleep stages. Five layers of CNN were used to extract features from EEG data, then two transformers were used to encode the current and previous epochs. In addition, a large dataset with 2274 participants was used to train and evaluate the proposed model that achieved high accuracy. The disadvantage of this model is that the training of the second transformer requires a successive number of epochs (i.e., seven epochs).

Using the EEG signal in addition to time-frequency images was proposed in [25]. The authors proposed a sequence-to-sequence model able to learn from both EEG signals and images of time-frequency. This model utilized a CNN to extract features from raw signals and a bidirectional RNN to extract time–frequency features. In addition, they designed this model to take into consideration the complementary effect of both network streams (i.e., CNN and RNN) and the avoidance of overfitting. Although they achieved a high accuracy of sleep staging classification over five datasets, using two-stream networks consumes more time and compactional resources.

Ref. [26] proposed using a multi-level fusion technique over EEG and electromyography (EMG) data. The authors tried one level of fusion (i.e., data, features, and decision fusion) and found that multi-level fusion achieved better accuracy. They suggested a fusion technique constructed with the CNN and suited to the properties of various EEG and EMG inputs. In addition, they tested the proposed method over the Sleep-EDF dataset using three layers of CNN to extract features from Fpz-Cz, Pz-Oz, and EMG data. Their methods achieved high accuracy (i.e., 87.3%) and improved the N1 stage accuracy.

Very limited methods have been applied to children’s EEG signals. In [27], a deep neural network model was proposed for classifying children’s sleep stages. The proposed model utilized a modularized architecture that enables the neural network to have many layers without being constrained by the increasing number of hyperparameters. In addition, a dataset containing a sleep study of 344 patients was developed to train and evaluate the proposed system. The age of patients was between 2 and 18 years. Furthermore, the system achieved an accuracy of 83.36% over five stages of classification. The limitations of this system are its low accuracy for N1 and the lack of results from children under 2 years old.

A stacked 1D CNN- and LSTM-based method has been introduced in [13] and applied to a small private dataset of 26 children ranging from 2–12 years old. The method employed data from a single EEG channel and trained using the edge AI paradigm. Six EEG channels were explored, namely F3-M2, F4-M1, C3-M2, C4-M1, O1-M2, and O2-M1. The best F1 score of 76.5 was achieved using the F4-M1 channel.

It has been noted that most of the literature work used a small publicly available dataset, such as sleep-EDF [28], or a large private dataset. In addition, most of the experiments have been conducted using an adult dataset and applied to limited EEG channels.

In this study, a large-scale dataset is used with a wide age range of participants, and seven different EEG channels are explored.

## 3. Method

### 3.1. Dataset

**Figure 1:**
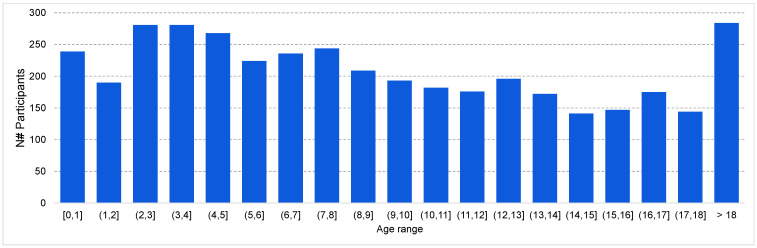
Distribution of the NCH dataset over different age ranges.

The Nationwide Children’s Hospital (NCH) dataset was created to cover the lack of pediatric publicly available sleep studies [29]. It has sleep recordings from 3984 patients collected in a clinical environment, where most of them are children. Figure 1 shows the number of participants in each age range. 

Approximately 92.56% of the patients have a single night’s recordings, while the rest have recordings from two or more nights, with a maximum of four. Of the participants, 56.3% were male and 43.7% were female. The total duration of the recordings is 40,884 h, with ~80% sampled at 256 Hz, ~14% at 400 Hz, and the remaining at 512 Hz.

The annotation of the sleep studies was conducted in two phases: a real-time phase, where a first technician annotated the sleep study during the sleep time of every participant, followed by an offline phase, where a second technician revised and corrected the annotation.

The dataset was split into 19 subsets on the basis of the age range of the participants. That is, the first group contains all recordings of participants 1 year old and younger, the second group contains all recordings of participants with ages between 1 and 2 years, and so on. The final group contains recordings of participants 18 years old and older. Participants in each group were further split into training, validation, and testing subsets, as explained in Section 4.

### 3.2. Sleep Staging Algorithms

Two sleep staging algorithms were utilized in this study, DeepSleepNet [20] and AttnSleep [18]. DeepSleepNet was chosen, as it is widely accepted and has been used as a benchmark in several recent works [19,30,31,32,33], while AttnSleep is an SOTA attention-based [34] method that was proven to outperform other existing work. Both algorithms are end-to-end deep learning-based with a trainable feature extraction network. The official implementations of both algorithms are available as open-source code, which we used without any modification in our experiments (https://github.com/akaraspt/deepsleepnet (accessed on 20 January 2022)) (https://github.com/emadeldeen24/AttnSleep (accessed on 18 February 2022)). In this section, an overview of the two algorithms is provided.

#### 3.2.1. DeepSleepNet

**Figure 2:**
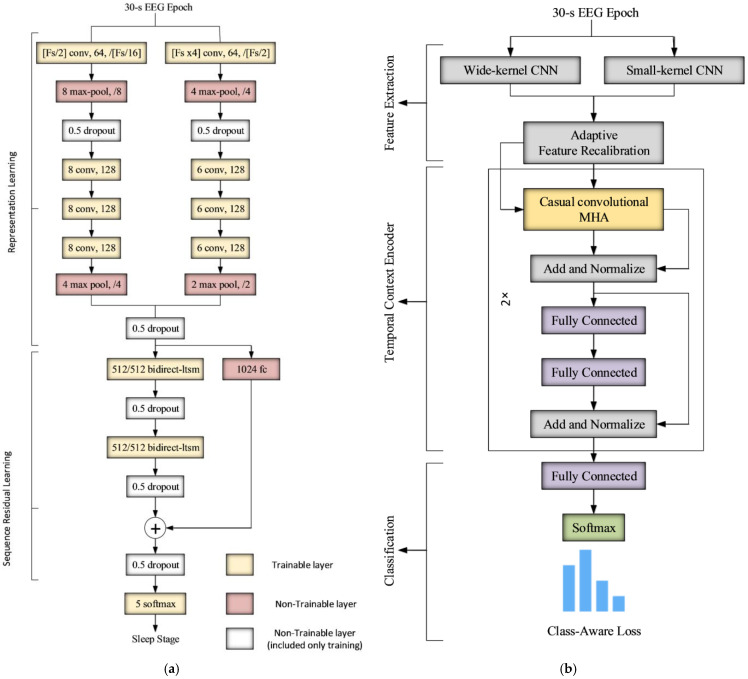
The architecture of the (**a**) DeepSleepNet and (**b**) AttnSleep sleep stage scoring systems.

In DeepSleepNet, time-invariant characteristics are extracted using CNNs, and transitions between sleep stages are learned using bidirectional LSTMs. The model architecture is shown in Figure 2a. In the first part, a multi-resolution CNN-based feature extraction module is deployed that consists of two CNNs with different filter sizes to capture high- and low-frequency components in the EEG signal. Each CNN has four layers of convolution and two layers of max-pooling. The outputs of both CNNs are then concatenated into one feature vector that inputs to the LSTM module. The LSTM module consists of two BLSTMs layers to learn to model sleep experts’ behaviour in detecting the next sleep stage on the basis of the previous stages.

A two-step training algorithm was proposed to train the DeepSleepNet model as an end-to-end using backpropagation to handle the class imbalance problem that usually exists in large datasets. The approach initially performs a supervised training of the first part of the model (i.e., CNNs) by connecting a softmax layer directly after the feature extraction network using a class-balance training set (i.e., all sleep stages are equally represented), which has been achieved by duplicating the minority sleep stages. The output softmax layer is then discarded and the pre-trained feature extraction network is connected to the LSTM network. Sequential training is then performed to fine-tune the entire model using an imbalanced training set. In both steps, the cross-entropy loss is used to measure the agreement between the anticipated and the desired sleep stages.

#### 3.2.2. AttnSleep

The AttnSleep algorithm adopts a multi-resolution CNN architecture similar to the DeepSleepNet as a feature extraction front-end network with an additional adaptive feature recalibration (AFR) network that acts as a feature selection module. 

Unlike the DeepSleepNet, the AttnSleep algorithm models the temporal contextual dependencies by adopting the attention mechanism [34]. The attention network consists of a multi-head attention (MHA) network followed by two “add and normalize” layers and two fully connected feed-forward layers. This architecture is similar to the transform block introduced in [34], with the exception that the positional encoding in the AttnSleep is achieved by a causal 1D convolutional layer instead of the sinusoidal positional encoding mechanism. The architecture is depicted in Figure 2b.

The class imbalance problem is handled in AttnSleep by introducing a class-aware loss function. The class-aware loss function is a weighted version of the cross-entropy loss function, where the loss of each class is scaled by a factor proportional to the number of training samples available for each class.

## 4. Experimental Setup

As previously mentioned, the main objective of this paper is to study the performance of the single EEG channel-based sleep staging algorithms over different types of channels and different subjects’ age ranges. Several experiments were conducted to better understand the impact of the type of EEG channel and the age of the participant on automatic sleep staging. All experiments have been performed using the two sleep staging algorithms detailed above, DeepSleepNet and AttnSleep.

**Figure 3:**
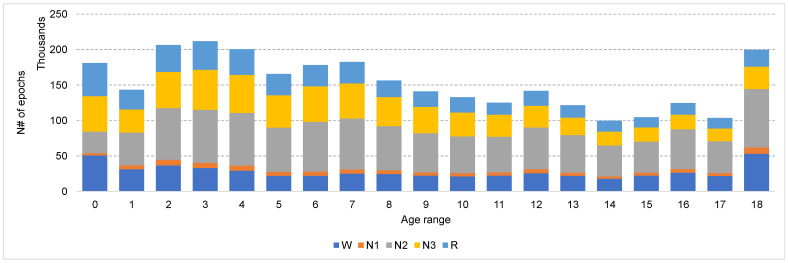
The distribution of the training data over different stages and age ranges.

The dataset is first divided into 19 subsets representing different age groups with a one-year step. That is, group 0 contains data from subjects aged from 6 days to 52 weeks, while group 19 contains data from subjects aged 18 years and over. Subjects of each age group are further split into training, validation, and testing sets. Of the subjects, 80% were used for training, while the other 20% was split equally between the validation and testing subsets. All three subsets had the same male/female distribution of subjects. Figure 3 shows the number of training epochs in each age range.

Seven EEG channels were employed in this study, namely C3-M2, O1-M2, O2-M1, CZ-O1, C4-M1, F4-M1, and F3-M2, as they are available in the recordings of ~99% of the participants [29]. For each EEG channel, recordings of participants from each age group were used to train two automatic sleep staging models, one for each automatic sleep staging algorithm. Given the seven channels and the 19 age groups, a total number of 126 models for each algorithm were trained. All models were trained to classify each epoch of 30 s duration as one of the five sleep stages, W, N1, N2, N3, and REM.

The parameters of the CNN feature extraction layers are the same in both algorithms, as they are a function of the sampling rate (see Figure 2). The parameters of the classification network and the training parameters are empirically determined, and the best values are listed in Table 1.

The sampling rate of the recordings was fixed to 256 Hz, and recordings with higher sampling rates were downsampled to 256 Hz. Both algorithms receive the raw EEG signal of a single epoch of 30 s as an input at each time step.

The F1 score was used to evaluate the classification performance of each stage i as follows:

(1) $$F1_{i}=\frac{2\times precison_{i}\times recall_{i}}{precision_{i}\times recall_{i}\;}$$

The overall performance was reported as a weighted average of the F1 scores of all stages as follows:

(2) $$WF1=\frac{1}{N}\underset{i}{\sum }S_{i}\times F1_{i}$$

where $S_{i}$ is the number of test samples of the stage i, and N is the total number of samples in the test set.

## 5. Results

### 5.1. Channel Analysis

As discussed, to investigate the impact of EEG channels on the accuracy of automatic sleep staging, seven DeepSleepNet models and seven AttnSleep models were trained and tested using data from one of the EEG channels.

**Figure 4:**
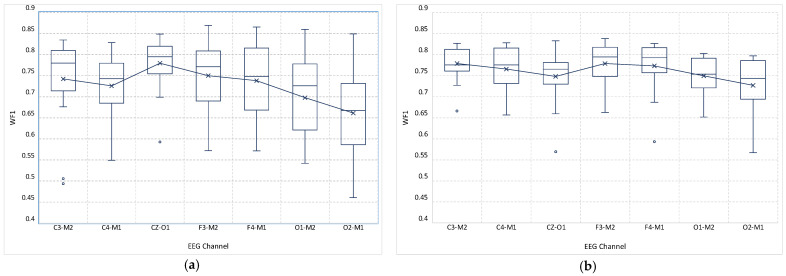
Box plot of the average WF1 of each EEG channel over all age groups for (**a**) DeepSleepNet and (**b**) AttnSleep algorithms. AttnSleep achieved higher average *WF*1, and less performance variation over different age groups compared to DeepSleepNet.

Figure 4a,b show the average WF1 of each channel over all age groups for both DeepSleepNet and AttnSleep, respectively. Overall, AttnSleep achieved a higher average *WF*1 compared with DeepSleepNet overall EEG channels, which is in line with the performance reported in [18] on other datasets. For AttnSleep, the highest average *WF*1 of ~77.8% was achieved on C3-M2 and F3-M2, followed by F4-M1, with an average *WF*1 of ~77.3%. On the other hand, with DeepSleepNet, the highest average WF1 of ~78% was obtained on CZ-O1, followed by F3-M2 and C3-M2, with average WF1 scores of ~75% and ~74.2%, respectively. For both algorithms, O1-M2 and O2-M1 channels obtained the lowest average WF1 scores of ~75% and ~72.7% for AttnSleep and ~70% and ~66% for DeepSleepNet, respectively.

As seen in Figure 4a,b, DeepSleepNet suffers from a high variation in performance over different age groups throughout all channels, with standard deviations ranging from ±5% on CZ-M1 to ±10% on O2-M1. On the contrary, AttnSleep was shown to have a more consistent performance over age groups, with standard deviations ranging from ±4% achieved on C3-M2 to ±6.6% achieved on O2-M1.

**Figure 5:**
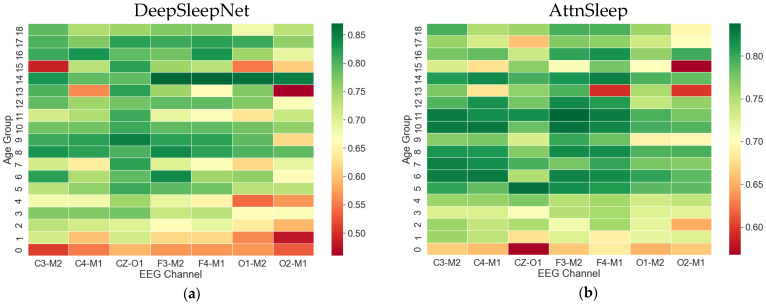
Heatmap of the WF1 scores of each channel over different age groups for (**a**) DeepSleepNet and (**b**) AttnSleep. In AttnSleep, F3-M2 and F4-M1 outperformed other channels in participants > 5 years old. In DeepSleepNet, CZ-O1 was superior in most age ranges.

Figure 5a,b show a heatmap of the WF1 scores of each channel over different age groups for the two adopted algorithms. AttnSleep performance on F3-M2 and F4-M1 was superior in subjects aged 5 years and older, while at younger ages, it outperformed on C3-M2 compared with other channels. With DeepSleepNet, the best performance was achieved on CZ-O1 over other channels in most age ranges.

**Figure 6:**
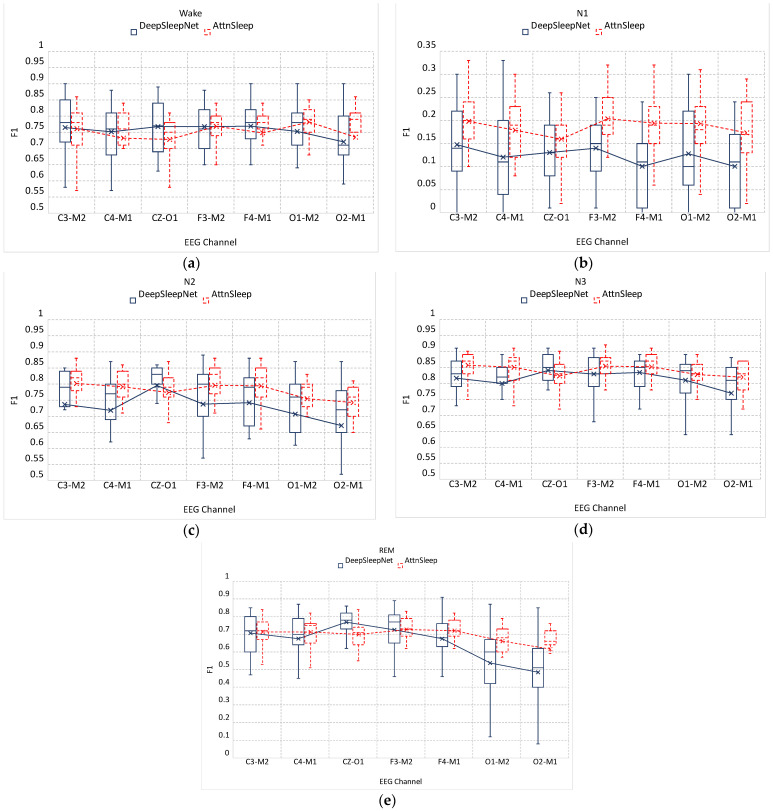
The DeepSleepNet (blue solid) and AttnSleep (red dashed) F1 scores over all age groups of the classification of the five sleep stages: (**a**) Wake, non-REM stages, (**b**) N1, (**c**) N2, (**d**) N3, and (**e**) REM. Overall, AttnSleep outperformed DeepSleepNet in the classification of almost all stages. N3 is the most accurate sleep stage while N1 is the most challenging one.

We further broke down the performance of the DeepSleepNet and AttnSleep algorithms into the five sleep stages, as depicted in Figure 6a–e. The performance is measured by the F1-score computed using Equation (1). N3 is the most accurate stage, with an average F1 score ranging from 85.6% ± 4% achieved on C3-M2 to 82% ± 4.3% achieved on the O2-M1 channel for the AttnSleep algorithm. For DeepSleepNet, the best average N3 detection F1 score of 84% ± 6.5% was obtained on CZ-O1, while the lowest average F1 score of 77% ± 13.3% was attained on O2-M1.

It is obvious from the figures that N1 is the most challenging stage, with an F1 score below 35% for both AttnSleep and DeepSleepNet models over all ages and channels. It is also noticeable that N1 has the highest performance variations among age groups in both algorithms compared with other stages.

### 5.2. Age Analysis

To explore the performance of automatic sleep staging at different age ranges, the NCH dataset was split into 19 groups on the basis of the age of the subjects from 0 to 18. The 0 group contains subjects aged up to 52 weeks, while the 18 group includes subjects of 18 years and older. Each model was trained and tested using data from the same age group.

In the previous section, CZ-O1 performed the best with DeepSleepNet and C3-M2 channel with AttnSleep. Therefore, in the following experiment, the CZ-O1 channel is used with the DeepSleepNet, and the C3-M2 channel is used with the AttnSleep.

**Figure 7:**
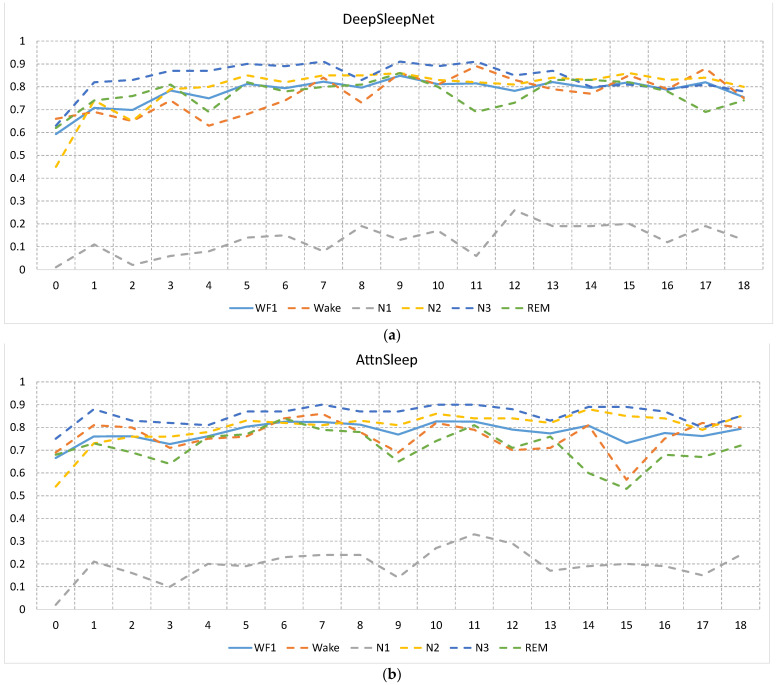
The F1 scores of the five sleep stages (dotted lines) over all age groups along with the WF1 score (solid line) for (**a**) DeepSleepNet and (**b**) AttnSleep. As seen, a noticeable degradation in the performance was occurred in infant participants (age group 0) in the classification of all stages.

Figure 7a,b show the F1 scores of the five sleep stages (dotted lines) over all age groups along with the WF1 score (solid line) for DeepSleepNet and AttnSleep algorithms, respectively. Each model was trained and tested using data from the same age group. As shown in the two figures, infant data (group 0) has the lowest F1 score over all stages and for both DeepSleepNet and AttnSleep algorithms compared with other age groups. The highest degradation in the performance of the infant data occurred in N1 and N2 stages, with F1 scores in the N1 stage close to 0% for both algorithms. 

For DeepSleepNet, the F1 score gradually increases with the age and is relatively saturated after group 5 (>5 years). On the other hand, N3 and N2 stages in AttnSleep were shown to be less affected by age, with the exception of infant (group 0), while REM and Wake suffered from inconsistency performance, with a high degradation occurring at ages 3, 9, and 14.

**Figure 8:**
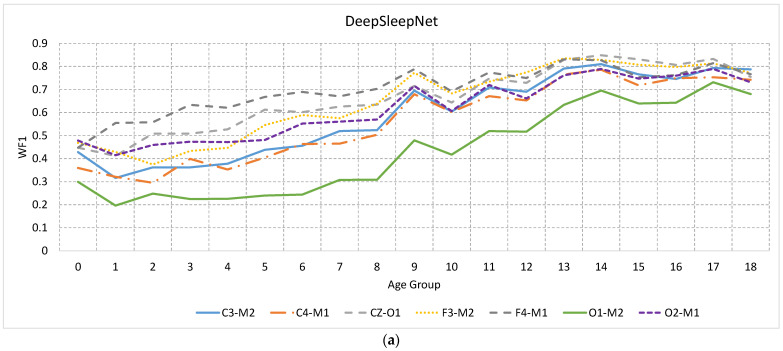

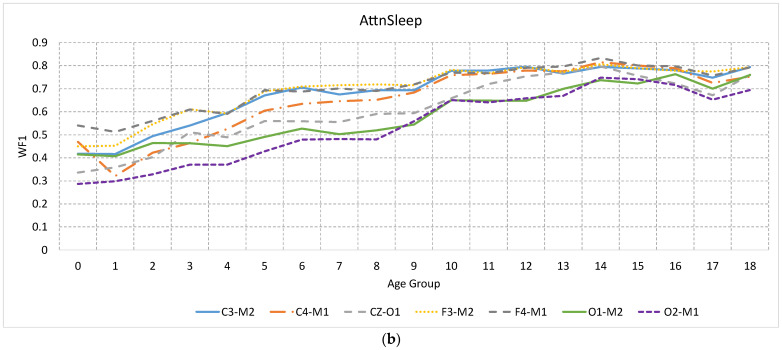
The performance of a model trained on adult EEG signal over different age ranges using different channels for (**a**) DeepSleepNet and (**b**) AttnSleep methods. As seen, the model had the lowest WF1 score when tested against infant EEG signal and increased gradually with the increase of the participants’ age.

As most of the available EEG datasets were collected from adult participants, such as the PhysioBank [28], Sleep-EDF, and UCD datasets, and SHHS [36], we tested the effectiveness of a model trained on adult data (>18) in detecting sleep stages of younger age groups. The results of the sleep staging of the two algorithms using the seven EEG channels over different age groups are depicted in Figure 8. The results clearly show that the performance of automatic sleep staging using the adult model improves gradually with age and starts to saturate after 11 years old and 14 years old for AttnSleep and DeepSleepNet, respectively. This is an important finding, demonstrating the development of the EEG signals with age and the cut-off age where these signals become more adult-like. In other words, there is a high degree of discrepancy between adult and child EEG signal at younger ages, which decreases gradually as the child grows up. Unlike the within-age group scenario where CZ-O1 and C3-M2 outperform other channels in DeepSleepNet and AttnSleep algorithms, respectively, here F4-M1 channel is superior to other channels in both algorithms.

**Figure 9:**
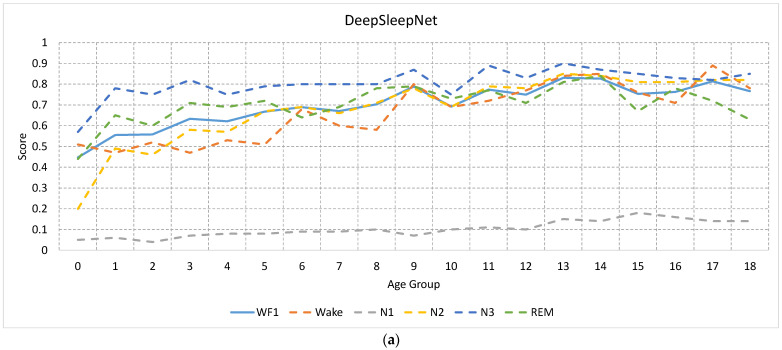

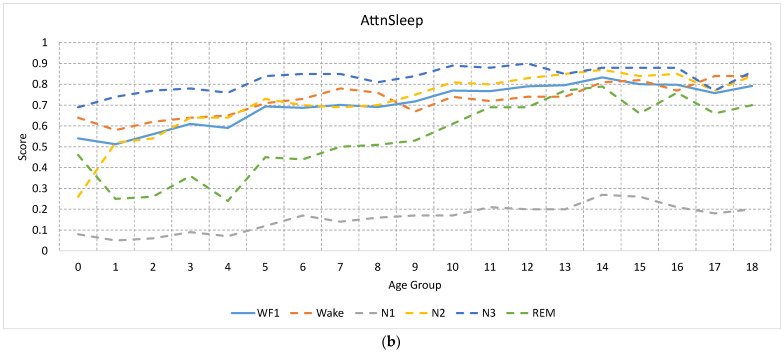
The breakdown of the adult model performance over different sleep stages for (**a**) DeepSleepNet and (**b**) AttnSleep.

Figure 9a,b show the breakdown of the performance over different sleep stages using the F4-M1 channel for AttnSleep and DeepSleepNet, respectively. In AttnSleep, the N3 stage was shown to be the stage least affected by the subject age, with an average F1 score of 82.7% ± 5.7%, while the REM stage has a significantly lower performance at younger ages compared with older ages, with an average F1 score of 54% ± 17%. 

Similarly, in DeepSleepNet, N3 has low variation in the performance between younger and older age groups compared with other stages, with an average F1 score of 80% ± 7%, while the N2 stage has a lower average F1 score of 69% and significantly higher standard deviation of ±17%.

## 6. Discussions

This paper provided, for the first time, an extensive comparison of the performance of automatic sleep staging of adults and children from a single EEG channel covering seven different EEG channels and a wide age range. The NCH recently released dataset was utilised for the study. The NCH dataset contains 40,884 h of sleep recordings collected from 3984 patients during a single night’s sleep. The participants were split into 19 groups on the basis of their age, from 0 (participants < 52 weeks) to 18 (participants >= 18 years old).

All experiments were conducted using two well-known deep learning-based automatic sleep staging algorithms, namely DeepSleepNet and AttnSleep.

A comparison of the two algorithms showed that AttnSleep performed better than the DeepSleepNet, specifically at younger ages (<2 years old) and also in the detection of the N1 stage throughout all ages (see Figure 6). Moreover, the performance of AttnSleep was more consistent over different ages compared with the DeepSleepNet, which suffers from high variations in performance among age groups. As explained in Section 3, both DeepSleepNet and AttnSleep adopt the same multi-resolution CNN front end module; however, AttnSleep architecture has an additional recalibration network AFR that works as an adaptive feature selection layer. As reported in the AttnSleep paper [18], this module is the most effective part of the network, as it has the ability to learn the inter-dependencies among features and adaptively select the most discriminative ones.

The channel analysis revealed that the models have the lowest average F1 score on O1-M2 and O2-M1 over almost all ages, indicating that these channels are not effective in training single-EEG channel automatic sleep staging models. The most effective three channels were C3-M2, F3-M2, and F4-M1 for AttnSleep and CZ-O1, F3-M2, and F4-M1 for DeepSleepNet, which have F3-M2 and F4-M1 in common (see Figure 4). Furthermore, F4-M1 was shown to be less affected by the age mismatch when used to train an adult model and tested with children’s data from different age groups (see Figure 8).

To demonstrate how significant the superiority of the model trained on a signal from the central and front EEG channels (C3-M2, CZ-O1, F3-M2, and F4-M1) was over the back channels (O1-M2 and O2-M1), we employed the almost stochastic order (ASO) significance test [37,38], as implemented by [39]. The method compares scores from two deep learning models A and B by computing the significance score $\epsilon_{min}$. If $\epsilon_{min}<0.5,$ then A is better than B based on a pre-defined significance level α. The lower $\epsilon_{min}$ is, the higher the confidence that A is better than B. Table 2 shows the significance score $\epsilon_{min}$ when applying the ASO test, with significance level α=0.05, to pairs of models trained on different EEG channels using both DeepSleepNet and AttnSleep algorithms. As shown in Table 2, $\epsilon_{min}<0.5$ for all channel pairs, which indicates that the models trained on central and front EEG channels data are significantly better than the models trained on back channels.

When the sleep staging models were trained and tested using data from the same age group, significant degradation in the performance of the detection of all stages for infant subjects (<52 weeks) was observed in both algorithms (see Figure 7).

When a model trained on adult data was used in performing sleep staging for child data, the performance was significantly lower in infant data and started to improve gradually to saturate after 13 years old (see Figure 8). Therefore, to achieve an accurate sleep staging for young subjects (<12 years old), the model should be trained on data from the age group, while an adult model can be reliable from 13 years old and older.

Overall, the N1 stage achieved the lowest classification accuracy compared with other stages in all experiments. This is a well-known problem that has been reported in several EEG-based sleep staging algorithms [18,20,30,40]. The possible reasons for this low classification accuracy are: Firstly, N1 is the shortest stage in the sleep cycle (~5%), which means that it has the lowest number of samples in the training set (see Figure 3), and therefore, even with the oversampling of the N1 class samples, the variations in the training data are not adequate to accurately model the N1 stage. Secondly, N1 is the transition stage between wake and deep sleep, and according to the AASM sleep-scoring guidelines [41], the characteristics of the EEG signal during the N1 stage is similar to those of the W stage, and therefore, high confusion between N1 and W stages occurs. Finally, Lee et. al. show in a recent study [42] that N1 has a very low interrater reliability of 0.24, while W, N2, N3, and R have interrater reliability of 0.7, 0.57, 0.57, and 0.69, respectively. The low interrater reliability affects the quality of the labels of the training data and, in turn, affects the accuracy of the trained model.

Compared with existing literature, to the best of our knowledge, no other work investigates the effect of age on the sleep staging classification performance. Moreover, the performance of the automatic sleep staging algorithm on pediatric sleep data has been briefly investigated. Jeon et al. [22] applied several deep and shallow machine learning methods to private pediatric data of children aged 10–15 years old and used multiple EEG channels. When using signals from 19 EEG channels, the average F1 score was 0.9 and dropped to 0.86 when the number of channels reached 6. In our study, the average F1 score of children of the same age range was ~0.8 when only one EEG channel was utilized. Although they used several EEG channels, they did not investigate which channel is the most effective but focused on the optimum number of channels to be used.

In [27] a CNN-based sleep staging model has been applied to private data collected from 344 children aged 2–18 years old. Although the data they used has a wide age range, the model was trained and tested using data from all age ranges, and the reported performance was averaged over all participants due to the lack of sufficient participants at each age group to train age-specific models.

Table 3 compares the results of the NCH dataset to the work performed on the Sleep-EDF and SHHS datasets using DeepSleepNet and AttnSleep algorithms. For a fair comparison, the results of NCH were obtained from the adult model (>18), as both Sleep-EDF and SHHS datasets were collected from adult participants. The results of the Sleep-EDF and SHHS datasets are obtained from the AttnSleep paper [18].

The NCH is shown to be more challenging than the other datasets; one possible reason is that most NCH participants have some type of sleep disorder, while other datasets are collected from participants with healthy sleep.

## 7. Conclusions

Intensive experiments were conducted using a recently released large-scale pediatric sleep dataset for the automatic sleep-scoring problem to investigate the impact of the type of the EEG channel and the patient’s age on the accuracy of the single EEG channel sleep scoring system.

Two recent and widely accepted algorithms of automatic sleep scoring were utilized in this work. Approximately 126 models were trained and evaluated covering 19 age groups and seven different EEG channels. The experimental results provide the following answers to the underlying research questions:

- How does the type of EEG channel used impact the accuracy of automatic sleep staging?

The experiments clearly showed that the performance of the automatic sleep scoring was significantly affected by the electrode position of the channel. The EEG signal obtained from the back electrodes (O1 and O2) achieved consistently lower accuracy compared with the central (Cz) and front (F3, F4) electrodes. A similar finding has been reported in [13], where six EEG channels were compared and O1-M2 and O2-M1 obtained the lowest F1 score of ~0.67 and ~0.65, respectively, while F4-M1 achieved the highest F1 score of 0.76. 

This finding suggests that when training a single EEG-based automatic sleep-scoring system, O1 and O2 should be avoided, and Cz, F3, and F4 are more likely to achieve higher classification accuracy.

- How does the accuracy of automatic sleep staging vary with the subject’s age?

A noticeable degradation in the accuracy of the infant participants using a model trained and tested on infant EEG signals occurred in both utilized algorithms. Elder ages achieved consistent performance when tested using models trained on a dataset of the same age group.

This finding suggests that, with the exception of infants, age-specific automatic sleep-scoring models, i.e., model trained and tested on a dataset from the same age group, are effective and achieved reasonable average classification accuracy over all age groups.

- How effective is a model trained on adult EEG data for sleep staging on pediatric EEG signals?

Experimental results showed that the classification accuracy of the sleep-scoring model trained on adult participants (>18 years) was maintained when tested on participants of 13 years and older. The accuracy of the adult model degraded gradually for ages younger than 13 years old, with the lowest accuracy obtained in participants in age groups 0 and 1 (<2 years).

This finding suggests that the model trained on adult EEG signals is not reliable for use in sleep scoring of younger ages. Training data from matching age groups is needed to achieve reasonable accuracy.

This work is considered the first work to study the influence of EEG channels and participants’ age using a large-scale dataset that includes participants as young as a few days old. However, the study is limited to one dataset and needs to be extended to other datasets to validate the generalization of the findings. Moreover, this study focuses on the performance of the single EEG channel sleep-scoring algorithms. However, using a multichannel (e.g., multiple EEG channels and EEG+EOG) model may improve the performance, specifically in younger ages.

Our future work includes investigating several domain adaptation techniques to alleviate the impact of age variations on the performance of the sleep-scoring algorithms.

## Figures and Tables

**Table 1:** Parameter settings of the DeepSleepNet and AttnSleep methods.

Parameter	DeepSleepNet	AttnSleep
No. epochs	100 (pre-training)/300 (fine-tuning)	100
Batch size	100	128
Optimizer	Adam [35]	Adam
Learning rate	0.0001	0.001
Weight decay	0.01	0.001
Dropout	0.5	0.5
No. attention heads	N/A	5
Feature dimension	N/A	100

**Table 2:** The ASO significance score $\epsilon_{min}$ of pairs of models A and B, where model A is trained on data from central and front EEG channels, and model B is trained on data from back channels. If $\epsilon_{min}<0.5$, then A is stochastically dominant over B. The lower the value of $\epsilon_{min}$, the higher the confidence that A is better than B.

		Model B			
		DeepSleepNet		AttnSleep	
		O1-M2	O2-M1	O1-M2	O2-M1
Model A	CZ-O1	0.05	0.008	0.21	0.04
	F3-M2	0.23	0.04	0.23	0.45
	F4-M1	0.36	0.07	0.23	0.07
	C3-M2	0.41	0.18	0.57	0.23

**Table 3:** Comparing NCH dataset (adult part) to the other sleep datasets in performing sleep stage scoring using DeepSleepNet and AttnSleep methods.

Dataset	Algorithm	No. Participants	EEG-Channel	F1 Score					Macro F1
				W	N1	N2	N3	REM	
Sleep-EDF-20	DeepSleepNet	20	Fpz-Cz	0.87	0.45	0.85	0.83	0.83	0.76
	AttnSleep		Fpz-Cz	0.9	0.42	0.89	0.9	0.79	0.78
Sleep-EDF-78	DeepSleepNet	78	Fpz-Cz	0.9	0.45	0.79	0.72	0.71	0.72
	AttnSleep		Fpz-Cz	0.92	0.42	0.85	0.82	0.74	0.75
SHHS	DeepSleepNet	329	C4-A1	0.85	0.4	0.82	0.79	0.81	0.74
	AttnSleep		C4-A1	0.87	0.33	0.87	0.87	0.82	0.75
NCH	DeepSleepNet	151 (>18)	C3-M2	0.81	0.24	0.83	0.81	0.72	0.68
	AttnSleep		F4-M2	0.84	0.3	0.84	0.85	0.72	0.7

## Data Availability

The NCH dataset (reference [29] in our text) used to support the findings of this study is free and available at https://sleepdata.org/datasets/nchsdb (accessed on 27 October 2021).

## References

[1] Murali N.S., Svatikova A., Somers V.K. (2003). Cardiovascular physiology and sleep. Front. Biosci.-Landmark.

[2] Yates P. (1987). Snoring as a risk factor for ischemic heart disease and stroke in men. Br. Med. J. (Clin. Res. Ed.).

[3] Baglioni C., Battagliese G., Feige B., Spiegelhalder K., Nissen C., Voderholzer U., Lombardo C., Riemann D. (2011). Insomnia as a predictor of depression: A meta-analytic evaluation of longitudinal epidemiological studies. J. Affect. Disord..

[4] Buxton O.M., Marcelli E. (2010). Short and long sleep are positively associated with obesity, diabetes, hypertension, and cardiovascular disease among adults in the United States. Soc. Sci. Med..

[5] Mendelson W. (2012). Human Sleep: Research and Clinical Care.

[6] Malhotra R.K., Avidan A.Y. (2013). Chapter 3—Sleep Stages and Scoring Technique. Atlas of Sleep Medicine.

[7] Zhu G., Li Y., Wen P. (2014). Analysis and classification of sleep stages based on difference visibility graphs from a single-channel EEG signal. IEEE J. Biomed. Health Inform..

[8] Fraiwan L., Lweesy K., Khasawneh N., Wenz H., Dickhaus H. (2012). Automated sleep stage identification system based on time—Frequency analysis of a single EEG channel and random forest classifier. Comput. Methods Programs Biomed..

[9] Şen B., Peker M., Çavuşoğlu A., Çelebi F.V. (2014). A comparative study on classification of sleep stage based on EEG signals using feature selection and classification algorithms. J. Med. Syst..

[10] Roy Y., Banville H., Albuquerque I., Gramfort A., Falk T.H., Faubert J. (2019). Deep learning-based electroencephalography analysis: A systematic review. J. Neural Eng..

[11] Tsinalis O., Matthews P.M., Guo Y., Zafeiriou S. (2016). Automatic sleep stage scoring with single-channel EEG using convolutional neural networks. arXiv.

[12] Fu M., Wang Y., Chen Z., Li J., Xu F., Liu X., Hou F. (2021). Deep learning in automatic sleep staging with a single channel electroencephalography. Front. Physiol..

[13] Zhu L., Wang C., He Z., Zhang Y. (2022). A lightweight automatic sleep staging method for children using single-channel EEG based on edge artificial intelligence. World Wide Web.

[14] Zhao S., Long F., Wei X., Ni X., Wang H., Wei B. (2022). Evaluation of a Single-Channel EEG-Based Sleep Staging Algorithm. Int. J. Environ. Res. Public Health.

[15] Urtnasan E., Park J.-U., Joo E.Y., Lee K.-J. (2022). Deep Convolutional Recurrent Model for Automatic Scoring Sleep Stages Based on Single-Lead ECG Signal. Diagnostics.

[16] Li Y., Peng C., Zhang Y., Zhang Y., Lo B. (2022). Adversarial learning for semi-supervised pediatric sleep staging with single-EEG channel. Methods.

[17] Li T., Zhang B., Lv H., Hu S., Xu Z., Tuergong Y. (2022). CAttSleepNet: Automatic End-to-End Sleep Staging Using Attention-Based Deep Neural Networks on Single-Channel EEG. Int. J. Environ. Res. Public Health.

[18] Eldele E., Chen Z., Liu C., Wu M., Kwoh C.K., Li X., Guan C. (2021). An Attention-Based Deep Learning Approach for Sleep Stage Classification With Single-Channel EEG. IEEE Trans. Neural Syst. Rehabil. Eng..

[19] Mousavi Z., Rezaii T.Y., Sheykhivand S., Farzamnia A., Razavi S. (2019). Deep convolutional neural network for classification of sleep stages from single-channel EEG signals. J. Neurosci. Methods.

[20] Supratak A., Dong H., Wu C., Guo Y. (2017). DeepSleepNet: A model for automatic sleep stage scoring based on raw single-channel EEG. IEEE Trans. Neural Syst. Rehabil. Eng..

[21] Kryger M.H., Roth T., Dement W.C. (2010). Principles and Practice of Sleep Medicine E-Book.

[22] Jeon Y., Kim S., Choi H.-S., Chung Y.G., Choi S.A., Kim H., Yoon S., Hwang H., Kim K.J. (2019). Pediatric sleep stage classification using multi-domain hybrid neural networks. IEEE Access.

[23] Ghimatgar H., Kazemi K., Helfroush M.S., Aarabi A. (2019). An automatic single-channel EEG-based sleep stage scoring method based on hidden Markov Model. J. Neurosci. Methods.

[24] Kim D., Lee J., Woo Y., Jeong J., Kim C., Kim D.-K. (2022). Deep Learning Application to Clinical Decision Support System in Sleep Stage Classification. J. Pers. Med..

[25] Phan H., Chén O.Y., Tran M.C., Koch P., Mertins A., De Vos M. (2021). XSleepNet: Multi-view sequential model for automatic sleep staging. IEEE Trans. Pattern Anal. Mach. Intell..

[26] Kim H., Lee S.M., Choi S. (2022). Automatic sleep stages classification using multi-level fusion. Biomed. Eng. Lett..

[27] Wang H., Lin G., Li Y., Zhang X., Xu W., Wang X., Han D. (2021). Automatic Sleep Stage Classification of Children with Sleep-Disordered Breathing Using the Modularized Network. Nat. Sci. Sleep.

[28] Goldberger A.L., Amaral L.A., Glass L., Hausdorff J.M., Ivanov P.C., Mark R.G., Mietus J.E., Moody G.B., Peng C.-K., Stanley H.E. (2000). PhysioBank, PhysioToolkit, and PhysioNet: Components of a new research resource for complex physiologic signals. Circulation.

[29] Lee H., Li B., DeForte S., Splaingard M.L., Huang Y., Chi Y., Linwood S.L. (2022). A large collection of real-world pediatric sleep studies. Sci. Data.

[30] Sors A., Bonnet S., Mirek S., Vercueil L., Payen J.-F. (2018). A convolutional neural network for sleep stage scoring from raw single-channel EEG. Biomed. Signal Process. Control.

[31] Fatimah B., Singhal A., Singh P. (2022). A multi-modal assessment of sleep stages using adaptive Fourier decomposition and machine learning. Comput. Biol. Med..

[32] Sokolovsky M., Guerrero F., Paisarnsrisomsuk S., Ruiz C., Alvarez S.A. (2019). Deep learning for automated feature discovery and classification of sleep stages. IEEE/ACM Trans. Comput. Biol. Bioinform..

[33] Phan H., Andreotti F., Cooray N., Chén O.Y., De Vos M. (2019). SeqSleepNet: End-to-end hierarchical recurrent neural network for sequence-to-sequence automatic sleep staging. IEEE Trans. Neural Syst. Rehabil. Eng..

[34] Vaswani A., Shazeer N., Parmar N., Uszkoreit J., Jones L., Gomez A.N., Kaiser Ł., Polosukhin I. (2017). Attention is all you need. Adv. Neural Inf. Process. Syst..

[35] Kingma D.P., Ba J. (2014). Adam: A method for stochastic optimization. arXiv.

[36] Zhang G.-Q., Cui L., Mueller R., Tao S., Kim M., Rueschman M., Mariani S., Mobley D., Redline S. (2018). The National Sleep Research Resource: Towards a sleep data commons. J. Am. Med. Inform. Assoc..

[37] Dror R., Shlomov S., Reichart R. Deep Dominance—How to Properly Compare Deep Neural Models. Proceedings of the 57th Annual Meeting of the Association for Computational Linguistics.

[38] Del Barrio E., Cuesta-Albertos J.A., Matrán C. (2018). An Optimal Transportation Approach for Assessing Almost Stochastic Order. Math. Uncertain.

[39] Ulmer D., Hardmeier C., Frellsen J. (2022). Deep-significance-Easy and Meaningful Statistical Significance Testing in the Age of Neural Networks. arXiv.

[40] Mousavi S., Afghah F., Acharya U.R. (2019). SleepEEGNet: Automated sleep stage scoring with sequence to sequence deep learning approach. PloS One.

[41] Berry R.B., Brooks R., Gamaldo C.E., Harding S.M., Marcus C., Vaughn B.V. (2012). The AASM manual for the scoring of sleep and associated events. Rules, Terminology and Technical Specifications.

[42] Lee Y.J., Lee J.Y., Cho J.H., Choi J.H. (2022). Interrater reliability of sleep stage scoring: A meta-analysis. J. Clin. Sleep Med..

